# The Role of Protein Tyrosine Phosphatase (PTP)-1B in Cardiovascular Disease and Its Interplay with Insulin Resistance

**DOI:** 10.3390/biom9070286

**Published:** 2019-07-17

**Authors:** Shahenda S. Abdelsalam, Hesham M. Korashy, Asad Zeidan, Abdelali Agouni

**Affiliations:** 1 Department of Pharmaceutical Sciences, College of Pharmacy, QU health, Qatar University, P.O. Box 2713, Doha, Qatar; 2 Department of Basic Sciences, College of Medicine, QU health, Qatar University, P.O. Box 2713, Doha, Qatar

**Keywords:** PTP1B, cardiovascular disease, endothelial dysfunction, insulin resistance, diabetes

## Abstract

Endothelial dysfunction is a key feature of cardiovascular disorders associated with obesity and diabetes. Several studies identified protein tyrosine phosphatase (PTP)-1B, a member of the PTP superfamily, as a major negative regulator for insulin receptor signaling and a novel molecular player in endothelial dysfunction and cardiovascular disease. Unlike other anti-diabetic approaches, genetic deletion or pharmacological inhibition of PTP1B was found to improve glucose homeostasis and insulin signaling without causing lipid buildup in the liver, which represents an advantage over existing therapies. Furthermore, PTP1B was reported to contribute to cardiovascular disturbances, at various molecular levels, which places this enzyme as a unique single therapeutic target for both diabetes and cardiovascular disorders. Synthesizing selective small molecule inhibitors for PTP1B is faced with multiple challenges linked to its similarity of sequence with other PTPs; however, overcoming these challenges would pave the way for novel approaches to treat diabetes and its concurrent cardiovascular complications. In this review article, we summarized the major roles of PTP1B in cardiovascular disease with special emphasis on endothelial dysfunction and its interplay with insulin resistance. Furthermore, we discussed some of the major challenges hindering the synthesis of selective inhibitors for PTP1B.

## 1. Introduction

In the past 20 years, obesity has become a global epidemic affecting both adults and children. The high increase in the number of cases of obesity had detrimental effects on morbidity and mortality and drove the social and healthcare costs to unprecedented levels. In a recent report by the World Health organization (WHO), it was estimated that around two billion individuals (39%), aged 18-years-old and above, are overweight, of which 650 million accounts for obese individuals. On the whole, obese individuals represent 13% of the world’s adult population [1]. This situation has contributed to reducing life expectancy of obese individuals [2,3,4].

The obesity epidemic is the consequence of a combination of effects, which include genetic predisposition, high intake of fat-rich diets and decreased physical activity due to rapid urbanization and sedentary lifestyle [3]. Very alarming is the concurrent rise in the prevalence of obesity-related pathologic conditions (Table 1), such as metabolic syndrome, type-2 diabetes mellitus and concurrent cardiovascular complications and certain forms of cancer [5].

Of particular interest, diabetes mellitus is a prevalent metabolic disease that occurs as an outcome of deficient insulin secretion and/or action, and persists to be a leading global health problem [6]. In 2017, the international federation of diabetes (IDF) has estimated that currently there are 425 million people affected with diabetes worldwide. It is predicted that over the next 28 years the number of diabetic patients will rise by 48%, reaching a staggering 629 million by 2045 [7]. Microvascular and macrovascular disturbances are key complications of diabetes, leading to failure of multiple organs including, kidneys, heart, the peripheral nervous system and cardiovascular tree [6] placing diabetes as the main cause of loss of vision and chronic kidney disease. Moreover, diabetes is associated with elevated risk for myocardial infarction and ischemic stroke [8,9]. In 2016, diabetes directly caused around 1.6 million deaths worldwide [10] and this number has later increased to four million in 2017 [7].

Numerous studies have focused on the link between obesity, diabetes and cardiovascular diseases. Insulin occupies a pivotal role in the metabolism of carbohydrates and lipids in the peripheral system, in addition to other important functions in the heart, vessels and the brain [11]. Hence, insulin resistance is associated with excessive influx of fatty acids and a pro-inflammatory state characterized by the overproduction of cytokines from adipocytes and infiltrating monocytes and macrophages, contributing largely to the pathophysiology of obesity and diabetes. Thus, detrimental cardiovascular alterations are the consequence of increased inflammatory factors and reactive oxygen species (ROS). Diabetes induced-vascular dysfunction, particularly endothelial dysfunction, involves at several molecular levels insulin resistance [9]. Insulin mediates its actions on the vessels by stimulating the release of nitric oxide (NO) by endothelial cells through the activation of endothelial NO synthase (eNOS), the main enzyme producing NO in the endothelium. Insulin resistance states impair endothelium-induced vasodilation, a phenomenon known as endothelial dysfunction [12]. Reduced bioavailability of NO is a characteristic hallmark of endothelial dysfunction, which profoundly affects peripheral vascular resistance and blood and nutrients delivery to metabolically active tissues, thus contributing to metabolic abnormalities. Endothelial dysfunction is an independent predictor of future adverse cardiovascular events through its critical role in atherogenic process and development of cardiovascular complications linked to diabetes [13,14].

Insulin receptor (IR) is a tyrosine kinase, which is negatively controlled by protein tyrosine phosphatases (PTP), particularly PTP1B, which is expressed in key tissues that regulate glucose metabolism such as the liver, skeletal muscle and adipose tissue [15]. PTP1B has gained much attention in recent years due to its ability to attenuate insulin signaling and it is currently regarded as a potential therapeutic target against metabolic syndrome, obesity and diabetes. Several studies have demonstrated the role of PTP1B in the negative regulation of insulin sensitivity and metabolism using whole-body and tissue-specific PTP1B knockout mouse models [16]. Furthermore, PTP1B was found to contribute directly, or indirectly (through insulin resistance), to the impairment of cardiovascular functions particularly endothelial function.

In this review article, we have summarized the major roles of PTP1B in cardiovascular disease with special focus on endothelial dysfunction and its interplay with insulin resistance. Furthermore, we discussed some of the main challenges hindering the synthesis of selective inhibitors for PTP1B.

## 2. The Endothelium

The endothelium is composed of a single layer of closely interconnected cells that line the whole of the vasculature. It operates as a large endocrine organ, which secretes a variety of factors that control several important functions. As the interface between blood stream and adjacent tissues, the endothelium tightly controls the flow of molecules, nutrients and blood cells [13,17,18,19].

### 2.1. The Physiological Roles of the Endothelium

The endothelial lining acts as a semipermeable membrane forming a biological barrier between the intravascular and extravascular divisions and thereby regulating transport and exchange of molecules [20]. Through its secretory functions, endothelial cells have a major contribution in the regulation of hemodynamics and the maintenance of vascular tone [21]. The understanding of the importance of endothelial cells and their essential roles in vasoregulation was first established in 1980 by Furchgott and Zawadzki who found that isolated blood vessels relax in response to acetylcholine only in the presence of an intact endothelium [22]. Due to its position in contact with blood and circulatory molecules as well as the vascular wall cells, they are the first responders to stimuli and hence have an initial and central role in the development and progression of cardiovascular diseases [21]. The endothelium exerts its functions through the synthesis of several regulatory substances (Table 2), which are essential for maintaining a healthy vascular environment. Among the various substances released, NO is the most distinguished mediator [9,13,14,23].

**Figure 1:**
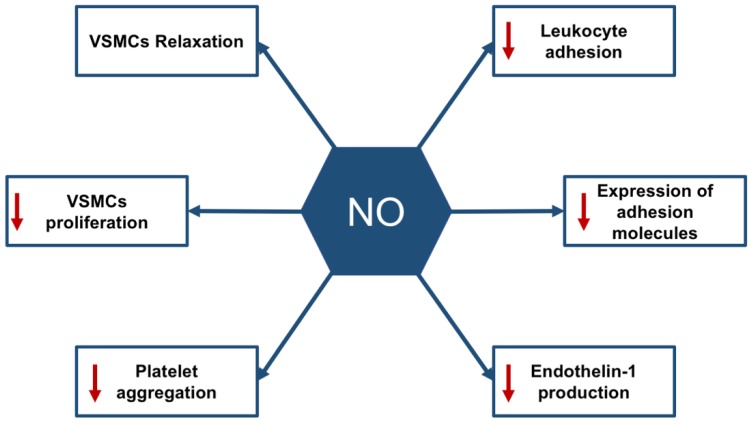
Nitric oxide (NO)-mediated physiological actions. NO has various physiological roles mediating endothelial function and maintaining vascular homeostasis as being a vasodilatory, anti-inflammatory and anti-coagulant factor. VSMCs—vascular smooth muscle cells.

Endothelial NO is synthesized by eNOS and released in response to several stimuli such as increased shear stress, as seen during physical exercise where vasodilation occurs as a result of vascular smooth muscle relaxation to increase blood flow. Besides relaxing vascular smooth muscle cells, NO also exerts anti-coagulant and anti-inflammatory functions (Figure 1). The anti-thrombotic effects of NO are mediated by its ability to prevent platelet aggregation and adhesion, in addition to promoting platelet disaggregation. Moreover, NO prohibits leukocytes’ adhesion to vessel walls along with the inhibition of migration and proliferation of vascular smooth muscle cells (VSMC). Furthermore, NO counteracts the prothrombotic effects of endothelin-1 (ET-1), a very potent vasoconstrictor synthesized by endothelial cells themselves [18,24].

**Figure 2:**
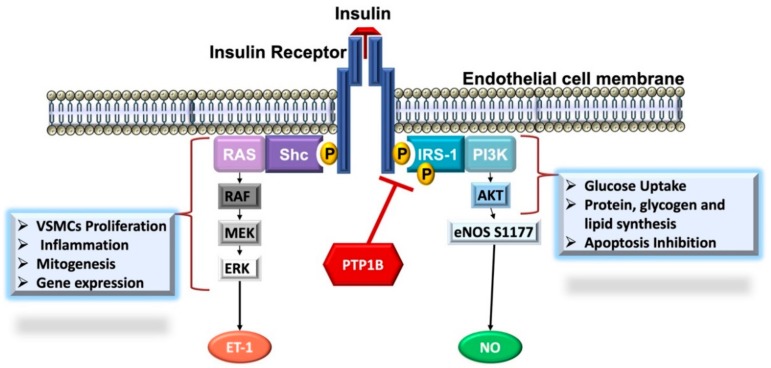
Insulin-mediated vascular actions. Insulin exerts vasodilatory and vasoconstricting actions in endothelial cells. Phosphatidylinositol-4,5-bisphosphate 3-kinase (PI3-K) branch of insulin signaling results in the phosphorylation (Ser1177) and subsequent activation of endothelial NO synthase (eNOS) stimulating NO production and release to induce vasodilation. Conversely, mitogen-activated protein kinase (MAPK) branch stimulates the production of endothelin-1 (ET-1) resulting in vasoconstriction. The negative regulatory actions of protein tyrosine phosphatase 1B (PTP1B) on insulin signaling response are indicated. Abbreviations: ERK, Extracellular signal-regulated kinases; MEK, MAPK ERK kinase, IRS-1, Insulin receptor substrate 1; Shc, Src homology domain.

Insulin exerts its vascular actions through the interaction with its receptor (IR) expressed by endothelial cells (Figure 2). The binding of insulin to IR on endothelial cells activates auto-phosphorylation of the receptor at tyrosine residues, which then phosphorylates IR substrate 1 (IRS-1) activating thus phosphoinositide-4,5-bisphosphate 3-kinase (PI3-K) signaling pathway, which culminates by the activation of Akt that can directly phosphorylate and hence activate eNOS, leading to an increase in NO yield. This action is followed by the diffusion of NO to the neighboring VSMCs and stimulates guanylate cyclase resulting in vasodilation [25,26].

### 2.2. Endothelial Dysfunction and Diabetes

As NO is an important mediator of the major functions of the endothelium, reduced NO production and release is a characteristic of impaired endothelial function. The endothelium becomes dysfunctional when it fails to maintain its physiological roles and shifts towards a vasoconstrictor, pro-thrombotic and/or pro-inflammatory state. Endothelial dysfunction is the initial step in atherogenesis and the progression of cardiovascular complications associated with diabetes [13,14,19]. A hallmark of metabolic disturbances occurring in diabetes include hyperglycemia, excess free fatty acids, hyperinsulinemia and insulin resistance. Insulin resistance can severely affect the function of endothelial cells and hence contribute to the progression of micro- and macrovascular complications observed in diabetes [25]. Okon et al. [27] found that arteries collected from diabetic individuals only exhibited half the phosphorylated levels of Akt compared to vessels obtained from controls, which supports the impairment of insulin signaling in vessels from diabetic patients. These observations were associated with impaired endothelial mediated-vasodilation [27]. Duncan et al. [28] generated transgenic mice specifically overexpressing a mutant of IR in endothelial cells and observed that these mice exhibited severely impaired endothelial function and reduced NO release [28].

Insulin exerts its action once it binds to IR, which undergoes auto-phosphorylation on tyrosine residues triggering thus the downstream signaling response. The insulin response is relayed via two major pathways; PI3-K/Akt pathway, which mediates most of insulin’s metabolic effects, such as glucose uptake and pro-survival actions, and the mitogen-activated protein kinase (MAPK) pathway, which along with the PI3-K govern cellular growth and differentiation. The activity of IR is finely controlled by the balance between its tyrosine phosphorylation and dephosphorylation. IR is negatively regulated by PTPs, particularly PTP1B [29]. PTP1B has gained much attention in recent years due to its ability to attenuate insulin signaling and is currently regarded as a possible therapeutic target against metabolic syndrome, obesity and diabetes [30].

## 3. Protein Tyrosine Phosphatase 1B

### 3.1. Protein Tyrosine Phosphatases Superfamily Family and PTP1B

**Figure 3:**
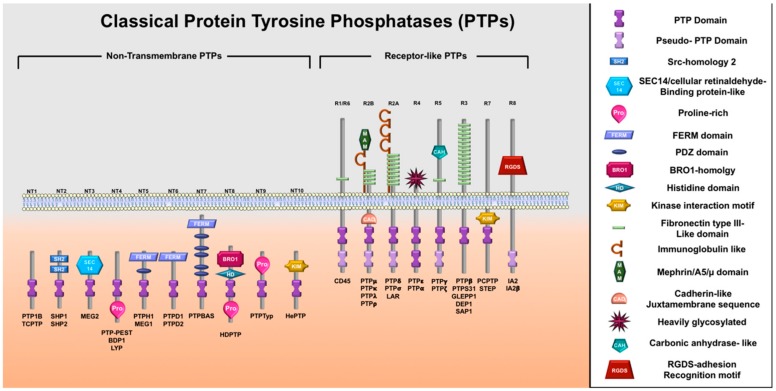
Classical PTPs. The classical PTPs are a large family of tyrosine phosphatases composed of 37 family members that are subdivided based on their cellular localization into 16 non-transmembrane (cytosolic) PTPs and 21 transmembrane receptor-like PTPs. The non-transmembrane phosphatases have one catalytic domain while receptor-like PTPs have either one or two catalytic domains, having the intrinsic catalytic cysteine residue.

PTPs are a large superfamily of enzymes composed of more than 100 different proteins, which their main function is to counteract the actions of protein tyrosine kinases (PTKs) [29]. A total of 125 PTPs have been determined in the human genome since the purification of the first PTP in 1988 [31]. This superfamily is characterized by its highly conserved catalytic domain, composed of around 240 amino acids having the active site hallmark motif C(X)5R [32], also referred to as Cys-X5-Arg [33] and (I/V)HCXAGXXR(S/T)G. The signature motif contains a catalytic cysteine that is paramount for the catalytic activity of the phosphatases, as it catalyzes the removal of phosphate moiety from the phosphotyrosine substrate [31]. The superfamily is categorized into three classes depending on their protein tyrosine specificities; class I, II and III. Class I is composed of the classical PTPs, while class II incorporates the dual specificity PTPs; these enzymes are able to remove phosphate groups from a phosphotyrosine as well as phosphoserine and phosphothreonine residues. Lastly, class III includes low molecular weight PTPs (Figure 3).

Class I is further sub-classified into two major sub-groups based on the enzymes’ cellular location; into receptor-like and cytosolic PTPs (Figure 3). The receptor-like PTPs consist of an extracellular receptor-like domain, a transmembrane domain and two cytoplasmic domains. Based on the features of the extracellular receptor-like domain in terms of size and sequence, receptor-like PTPs are further subdivided into five types. Cytosolic PTPs, also known as non-receptor like PTPs, are further divided into two subtypes; the classical tyrosine-specific phosphatases and dual-specific phosphatases. Tyrosine-specific phosphatases can dephosphorylate phosphotyrosine (p-Tyr)-containing proteins, while dual-specific phosphatases can hydrolyze proteins containing p-Tyr, as well as phosphoserine (p-Ser) and phosphothreonine (p-Thr). The depth of the active site pockets is ~9 A° and ~6 A°, for tyrosine-specific and dual-specific phosphatases, respectively [31,34].

Of particular interest and among the most important PTPs, there is PTP1B, which is a soluble non-transmembrane and cytosolic tyrosine-specific phosphatase. It represents the founding and most studied member of the PTP superfamily. It was first isolated from human placenta and characterized in 1988 by Tonks et al. [35,36]. Isolation of cDNA of human PTP1B showed that it consists of 435 amino acids and is 50 kDa in molecular size [37].

### 3.2. PTP1B Intracellular Localization and Molecular Substrates

PTP1B is a tail-anchored protein. Tail-anchored proteins have towards the C- terminus a single stretch transmembrane domain. After they are synthesized, they get anchored by the C-terminus to an intracellular membrane such as the endoplasmic reticulum (ER) or to the mitochondrial membrane [38]. PTP1B becomes anchored to the ER with the C-terminus 35 amino acid residues. These 35 residues are known to be hydrophobic in nature, which helps in directing the enzyme towards the cytoplasmic face of the ER membrane [39]. The anchoring of PTP1B to a specific subcellular location limits its access to substrates, restricting their interaction and hence plays a role in the regulation of its function. It was also shown that it contributes in the suppression of its catalytic activity; truncated PTP1B lacking around 75 residues from the C-terminus, including the ER-targeting motif, showed intensified catalytic activity [40]. PTP1B interacts with a diverse number of substrates that affect cellular physiology. PTP1B attenuates insulin signaling [41] as well as platelet-derived growth factor (PDGF), epithelial growth factor (EGF) and Janus kinase 2 (JAK2)/signal transducer and activator of transcription (STAT) signaling pathways [42] indicating the diverse roles of PTP1B in the regulation of different cellular processes.

With regards to endothelial function, little is known about the exact role of PTP1B in the regulation of eNOS activity and whether eNOS is a direct substrate for the tyrosine phosphatase action of PTP1B. It is well established that the activity of eNOS is regulated by phosphorylation on various residues chiefly on Ser1177 and Ser1179, which enhance its activity [43] and on Thr495 located in calmodulin binding domain, which decreases the activity of eNOS [44]. However, eNOS was also found to be phosphorylated on tyrosine residues in the presence of phosphatase inhibitors and in response to shear stress for instance [45,46]. eNOS is phosphorylated by non-receptor tyrosine kinase, c-Src, on Tyr83, which has been found to enhance eNOS activity following stimulation with various factors including acetylcholine and bradykinin [46,47]. Subsequently, eNOS was found to be phosphorylated in mice by proline-rich tyrosine kinase 2 on Tyr657, which was associated with a decrease in eNOS activity in endothelial cells challenged with angiotensin II [48] and cardiomyocytes exposed to hydrogen peroxide [49]. Despite some available data showing that PTP1B deletion or pharmacological inhibition is associated with enhanced activity of eNOS [50,51,52,53], it still not known whether PTP1B mediates its actions by direct dephosphorylation of eNOS on regulatory tyrosine residues such as Tyr83. Further work is warranted to establish the exact role of PTP1B in regulating the activity of eNOS.

### 3.3. The Involvement of PTP1B in the Pathophysiology of Cardio-Metabolic Diseases

#### 3.3.1. The Role of PTP1B in Obesity and Insulin Resistance

Insulin has a pivotal function in the metabolism of lipids and carbohydrates in liver, skeletal muscles and adipose tissue [11]. There is a direct interplay between obesity and diabetes evident by the high number of obese patients that develop diabetes as a consequence of weight gain. This results in a condition known as diabesity, a term that best describes the metabolic disturbances occurring in obese diabetic individuals. Patients showing diabesity share common disorders, such as endothelial and vascular dysfunction as well as an impairment in hepatic and adipose tissue homeostasis [54]. All these perturbations share a common feature of insulin resistance. Much evidence in the literature indicates that insulin resistance alone suffices to induce several of the components of the metabolic syndrome and initiates and maintains the progression to cardiovascular dysfunction in addition to metabolic disruption [55]. The role of PTP1B in insulin resistance was the main focus of several studies, showing that PTP1B targets tyrosine-phosphorylated IR and IRS-1, hence negatively attenuating their signal [56,57,58,59]. In the late 1990s, studies using whole-body PTP1B-deficient mice (PTP1B^−/−^) identified PTP1B as a master negative controller of body mass and insulin signaling pathway. PTP1B^−/−^ mice were more insulin sensitive in comparison to their wild type littermates. This was evident by their lower blood glucose concentrations accompanied by reduced levels of circulating insulin, which was 50% less than those observed in control mice. In addition, insulin-mediated signaling was greater in PTP1B^−/−^ mice as demonstrated by a two-fold increase in IR phosphorylation as compared to wild type mice [16]. Interestingly, when placed on a high fat diet (HFD), PTP1B^−/−^ mice were resistant to weight gain, lean and retained enhanced insulin sensitivity, while their littermates became obese and insulin resistant. Furthermore, PTP1B^−/−^ mice were protected against HFD-induced hepatic steatosis, while wild type control mice had excessive fat droplets accumulation in their livers [16,60]. In accordance with these findings, Klaman et al. [61] demonstrated that PTP1B^−/−^ mice were small, lean and gained less weight than their control counterparts. Even when fed a HFD, PTP1B^−/−^ mice continued to exhibit a lean profile. Moreover, PTP1B^−/−^ mice had reduced fat stores as observed by their (a) low fat pad mass, (b) a reduction in, not adipocytes number but size, and (c) elevated basal metabolic rate and energy expenditure [61].

**Figure 4:**
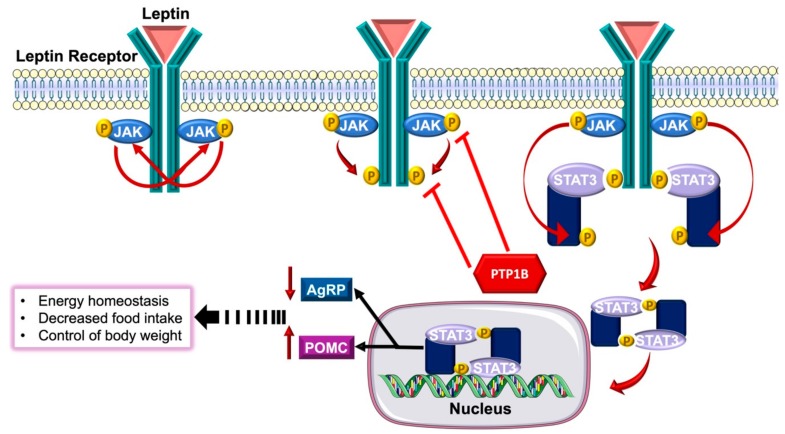
Leptin signaling. Binding of leptin results in the cross-linking of two adjacent receptors and the recruitment of Janus Kinase 2 (JAK2), which cross-phosphorylates each other. Activated JAKs phosphorylate tyrosine residues on the receptor allowing the docking of signal transducer and activator of transcription 3 (STAT3). JAKs phosphorylate STAT3, which then dissociate and dimerize. STAT3 dimers translocate to the nucleus where they upregulate the expression of anorexigenic protein pro-opiomelanocortin (POMC) and suppressing that of the orexigenic protein Agouti-related peptide (AgRP) mediating the regulation of energy expenditure, control of body weight and suppresses appetite. The negative regulatory actions of PTP1B on leptin signaling pathway are indicated.

The resistance of PTP1B^−/−^ mice to obesity and improved insulin sensitivity have led scientists to investigate the underlying molecular basis. One of the possible mechanisms proposed was the negative regulation of leptin receptor by PTP1B. Leptin is an anti-corpulence hormone derived from adipocytes and represents the key regulator of body mass through appetite suppression and elevation of energy expenditure [62]. Leptin receptor is ubiquitous in the central nervous system and is particularly abundant in the hypothalamus. Upon leptin interaction with its receptor, JAK2 is recruited, which phosphorylates several tyrosine residues on leptin receptor (Figure 4). This phosphorylation triggers a downstream signaling cascade mediating Signal transducer and activator of transcription 3 (STAT3) phosphorylation. This results into STAT3 dimerization and allowing it to subsequently translocate to the nucleus where it interacts with the main regulatory genes of appetite, boosting the transcription of the anorexigenic protein pro-opiomelanocortin (POMC) and suppressing that of the orexigenic protein Agouti-related protein (AgRP). As a result, appetite is suppressed, and energy expenditure is enhanced hence mediating leptin’s anti-obesity effect.

Perturbations in either the leptin protein or leptin receptor lead to morbid pathological obesity. Despite the elevated serum leptin levels found in obese individuals, yet it is unable to restore homeostasis, indicating the presence of leptin resistance. Several hypotheses were proposed to investigate the underlying mechanisms involved in this resistance, one of which is the negative regulatory effect of PTP1B [62]. Whole-body PTP1B^−/−^ mice injected with an exogenous dose of leptin exhibited hypersensitivity to leptin as well as enhanced leptin signaling in the hypothalamus. This enhancement was demonstrated through the upsurge of tyrosine phosphorylation of STAT3 by 35% in hypothalami collected from PTP1B^−/−^ mice compared to controls, indicating the role of PTP1B in regulating leptin signaling [63]. Double mutant mice were generated using leptin receptor mutant db/db mice and PTP1B^−/−^ mice. Leptin receptor mutant db/db mice are distinct by their morbid obesity, persistent state of insulin resistance and heightened levels of PTP1B. Deletion of PTP1B in db/db mice considerably enhanced glycemic control and ameliorated insulin sensitivity [64].

González et al. [65] have also used the concept of double mutants to further explore the interplay between PTP1B and insulin signaling. Mice deficient of IR substrate 2 (IRS-2^−/−^) had elevated blood glucose levels, insulin resistance and hepatic insulin signaling defects. This was accompanied by a rise in the levels of PTP1B expression and catalytic activity in the liver and pancreatic β-cells. In livers from control mice, insulin enhanced PI3-K signaling and phosphorylation of Akt; however, these effects were not observed in IRS-2^−/−^ mice. Fortunately, PTP1B deletion in IRS-2^−/−^ mice restored insulin-stimulated PI3-K activation and phosphorylation of Akt. Likewise, IRS-2^−/−^ mice failed to exhibit insulin-mediated phosphorylation of forkhead box protein O1 (FOXO1), a key regulatory protein of hepatic glucose production, which led to increased gluconeogenesis and hyperglycemia. On the other hand, double mutant mice displayed normal response to insulin. Altogether, PTP1B deletion in IRS-2^−/−^ mice restored glucose homeostasis and peripheral as well as hepatic insulin sensitivity [65].

As whole-body deletion of PTP1B was proven to be beneficial in improving global insulin sensitivity, glucose homeostasis and conferred resistance to HFD-induced obesity, the tissue responsible for PTP1B effects remained questionable. This has led to the generation of several tissue-specific PTP1B^−/−^ mice in the brain and in insulin-sensitive tissues such as skeletal muscle, liver and adipose tissue in addition to others. Deletion of PTP1B in the brain was shown to be the main mediator of weight gain resistance and reduced adiposity in whole-body PTP1B deficient mice, as demonstrated by work by Bence et al. [66], which reported that neuronal PTP1B^−/−^ mice maintained on a chow diet gained less body weight compared to their control counterparts; however, when challenged with a HFD, neuronal PTP1B^−/−^ mice had a similar body weight gain pattern to whole-body PTP1B^−/−^ mice. They also exhibited reduced food intake accompanied with an increase in energy expenditure. Exogenous leptin injection to neuronal PTP1B^−/−^ mice resulted in enhanced weight loss and appetite suppression evident by decreased food intake, indicating an enhancement of leptin sensitivity. In accordance with these findings, hypothalamic mRNA expression of AgRP was downregulated while POMC was upregulated. These effects were further enhanced by exogenous leptin injection. Moreover, neuronal PTP1B^−/−^ mice had enhanced glucose homeostasis, decreased serum levels of insulin and improved IR phosphorylation in both skeletal muscle and liver, illustrative of improved peripheral insulin sensitivity [66].

As POMC neurons are the principal mediators of leptin’s actions, POMC PTP1B^−/−^ mice were generated to assess whether the observed effects in neuronal PTP1B^−/−^ mice are mediated through POMC neurons. Similar to neuronal-specific PTP1B deletion, POMC-specific deletion of PTP1B rendered mice resistant to diet-induced obesity, increased energy expenditure, enhanced leptin and insulin sensitivity and improved glucose homeostasis [67]. These findings have led scientists to wonder to which extent PTP1B mediates its metabolic effects through leptin-expressing neurons, so leptin receptor-expressing neurons PTP1B^−/−^ (LepRb-PTP1B^−/−^) mice were generated. Similar outcomes were observed in LepRb-PTP1B^−/−^ consistent with whole-body, neuronal and POMC PTP1B^−/−^ mice, regarding the lean profile and enhancement of insulin and leptin sensitivities. Interestingly, on chow diet, whole-body PTP1B^−/−^ mice had less adiposity compared to controls while LepRb-PTP1B^−/−^ showed no difference, suggesting that PTP1B has additional metabolic roles that are independent of leptin [68].

All the previously mentioned studies emphasized on the role that PTP1B plays centrally. However, it was not clear what impact PTP1B has in peripheral tissues that regulate insulin function. Understanding the impact of PTP1B on peripheral tissues was very crucial from a drug targeting strategy point of view as it is more challenging to devise effective small molecules to target PTP1B in the brain compared to targeting peripheral tissues. Therefore, investigation of the impact PTP1B deletion on insulin signaling was carried on in mice with specific deletion of PTP1B in liver, muscle, adipose tissue and macrophages. Mice lacking PTP1B in skeletal muscle displayed similar body weight compared to their control littermates, both on HFD and chow diet. However, muscle-specific PTP1B^−/−^ mice had an improved glucose disposal compared to the controls, yet, without any changes in insulin levels, indicating the muscle specific deletion of PTP1B improved global insulin sensitivity [69]. On the other hand, liver-specific deletion of PTP1B resulted in an enhancement of insulin suppression of hepatic gluconeogenesis, where hepatic glucose production was significantly reduced in livers collected from HFD-fed liver-PTP1B^−/−^ mice compared to controls. Liver-PTP1B^−/−^ mice fed a HFD exhibited reduced levels of cholesterol along with a downregulation of the expression of genes responsible for its synthesis, namely sterol regulatory element–binding proteins (SREBP)-2 and 3-hydroxy- 3-methylglutaryl-coenzyme A synthase 1 (HMGCS1), in addition to decreased level of hepatic triglycerides [51,70]. Moreover, liver-PTP1B^−/−^ mice maintained on HFD presented with a decrease in the levels of fed glucose and circulating insulin, indicating an overall improvement of glucose homeostasis and hepatic insulin sensitivity [70,71]. A similar impact was observed in a mouse model of induced liver-specific PTP1B deletion in adult mice. Placed on HFD, adult liver-PTP1B^−/−^ mice displayed lower fed and fasted glucose levels, decreased hepatic glucose production, lessened serum levels of leptin in both fed and fasted states and a significant reduction in triglyceride levels in comparison to their control littermates [72]. These data in adult mice highlight the usefulness of PTP1B inhibition as a therapeutic approach in diabetes and insulin resistance after they were established.

On the contrary to all the beneficial effects of tissue-specific PTP1B deletion, adipose-specific deletion of PTP1B did not reveal any benefits in improving glucose or lipid homeostasis. In fact, adipose-PTP1B^−/−^ mice exhibited higher levels of blood glucose as well as circulating insulin levels compared to control. This was observed together with increased insulin resistance evident by decreased IR and Akt phosphorylation in adipocytes, indicating that PTP1B is not the main regulator of IR in adipose tissue [73].

Intriguingly, other studies have investigated the effect of tissue-specific over-expression of PTP1B. Mouse hypothalamic neuronal cell line, GT1-7, over-expressing PTP1B manifested a blunted leptin-induced tyrosine phosphorylation of JAK2 and STAT3. Over-expression of PTP1B resulted in the downregulation of genes, which are upregulated by leptin. Reciprocally, PTP1B over-expression elevated the expression of genes that are downregulated by leptin [74]. Zabolotony et al. [75] investigated the effects of PTP1B levels in transgenic mice overexpressing human PTP1B selectively in muscle at levels that resemble to those observed in cases of insulin resistance in humans. Mice displayed an impairment in insulin-induced glucose transport into skeletal muscle by 50% when compared controls, indicating that the over-expression of PTP1B in muscles led to insulin resistance [75]. Whole-body PTP1B^−/−^ mice had improved glucose uptake by the muscles as well as enhanced insulin signaling and phosphorylation of IR; however, those effects were blunted by liver re-expression of PTP1B in whole-body PTP1B^−/−^ mice [76].

#### 3.3.2. PTP1B and Cardiovascular Complications

##### PTP1B and Endothelial Dysfunction

Endothelial dysfunction is the primary event in the progression of cardiovascular complications, and the initial event in atherogenic process. It is a predictor of cardiovascular events in diabetes and obesity [77]. By attenuating insulin signaling and thus preventing insulin-mediated vascular effects, PTP1B has been found to play a major role in endothelial dysfunction and cardiovascular disorders. It is known that chronic heart failure (CHF) results in endothelial dysfunction demonstrated by diminished NO bioavailability and vasodilatory action. To investigate the implication of PTP1B, CHF-induced endothelial dysfunction in mice was used. CHF was brought about by ligating the main coronary artery. By increasing vascular blood flow, normal mice arteries displayed a reduction in flow-mediated dilation (FMD), while CHF arteries did not. PTP1B inhibition did not affect FMD in control mice; however, FMD was restored in arteries isolated from CHF mice. PTP1B inhibition also restored endothelial function as well as NO-induced FMD. The effects of CHF on impaired activatory phosphorylation of eNOS were mainly driven by oxidative stress, low grade inflammatory state and enhanced neuro-hormonal activity without altering the expression of PTP1B [53]. PTP1B deletion or its pharmacological inhibition protected mice against the adverse effects of CHF. Mice exhibited improved systolic pressure and reduced end-diastolic pressure along with enhanced cardiac output. This was accompanied by a reduction in ventricular remodeling, fibrosis and hypertrophy of cardiomyocytes. Using isolated mesenteric arteries from mice treated with either PTP1B inhibitor or obtained from PTP1B-deficient mice, it was revealed that an improvement in NO-mediated FMD as well as enhanced eNOS phosphorylation was observed in the absence of active PTP1B, indicating that PTP1B blockade improves endothelial function [52].

Aortas isolated from control and liver-PTP1B^−/−^ mice, both fed a HFD, revealed that HFD caused endothelial dysfunction in control mice, evident by impaired vasodilation in response to acetylcholine, while liver-specific PTP1B deletion protected mice against HFD-provoked endothelial dysfunction. In accordance, eNOS phosphorylation at Ser1177, which is eNOS activatory site, was significantly stimulated in aortas from liver-PTP1B^−/−^ mice fed a HFD, while it was reduced in control mice [51].

##### PTP1B and Vascular Inflammation

Inflammation is a major contributor to disturbances associated with obesity and diabetes. It heightens the risk of cardiovascular complications, specifically atherosclerosis [78]. Atherosclerosis, once considered as a lipid storage impairment, involves inflammatory processes throughout the disease stages [79]. Endothelial dysfunction precedes and triggers inflammation, where perturbed endothelial cells express at their surface several adhesion molecules such as vascular cell adhesion molecule 1 (VCAM-1) and intracellular molecular adhesion 1 (ICAM-1), accompanied by the production of various pro-inflammatory cytokines including TNF-α, interleukin (IL)-6 and IL-1β [80]. PTP1B has been reported to be involved in inflammatory responses [29]. Myeloid-PTP1B knockout mice were found to be protected against HFD and lipopolysaccharide (LPS)-induced inflammation. Macrophages isolated from myeloid-PTP1B knockout mice had decreased TNF-α mRNA levels while showing increased levels of the anti-inflammatory cytokine IL-10 [81]. Thompson et al. [82], have later generated a double knockout mouse model by crossing macrophage-specific PTP1B^−/−^ mice with apolipoprotein E (ApoE)-deficient mice that are prone to developing atherosclerosis. Double knockout mice (ApoE^−/−^ and macrophage-specific PTP1B^−/−^), had reduced plaque size and an improved glucose homeostasis in addition to enhanced plasma levels of IL-10 and prostaglandin E2 (PGE2) [82]. Similarly, the pharmacological inhibition of PTP1B in low-density lipoprotein receptor (LDLR)-deficient mouse model of atherosclerosis reduced plaque size and vascular expression of MCP-1 in addition to improving adiposity and insulin response [83]. These studies point out the potential of targeting PTP1B to improve vascular inflammation, a major contributor to atherogenesis. The role of PTP1B in inflammation was further harnessed in a cecal ligation and puncture sepsis mouse model, where PTP1B deletion prevented the enhanced release of TNF-α, IL-6 and IL-1β compared to wild type mice [84]. Further providing a clear link between PTP1B and inflammation, Zabolotny et al. [85], has shown that the overexpression of PTP1B was regulated by inflammation, where TNF-α increased PTP1B mRNA and protein expression through the activation of nuclear factor (NF)-κB pro-inflammatory transcription factor [85]. 

##### PTP1B and Altered Angiogenesis

**Figure 5:**
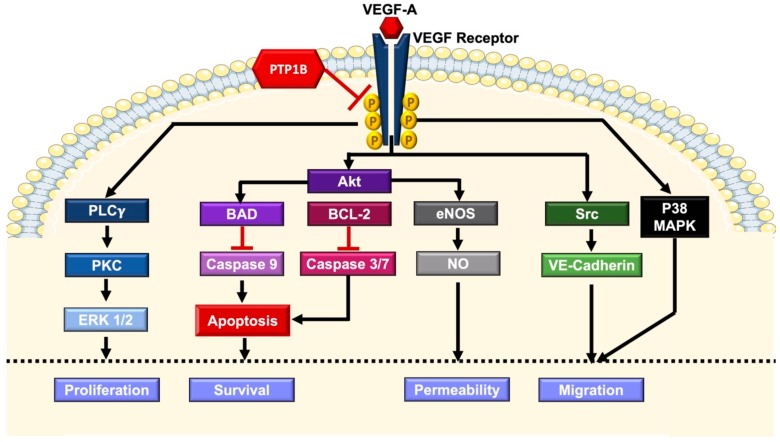
Vascular endothelial growth factor (VEGF) signaling. Binding of VEGF-A to its receptor induces the receptor’s intrinsic tyrosine kinase, which induces autophosphorylation initiating the downstream signaling cascade. The downstream effectors include, PLC_γ_/PKC/ERK 1/2, PI3-K/Akt and p38 MAPK, which induce endothelial cell proliferation, survival and migration, respectively. The negative regulatory actions of PTP1B are indicated. BCL-2, B-cell lymphoma 2; BAD, BCL-2 agonist of cell death; eNOS, endothelial NO synthase; PI3-K, Phosphatidylinositol-4,5-bisphosphate 3-kinase; PLC_γ_, phosphoinositide phospholipase C γ; PKC, Protein kinase C; ERK, Extracellular signal-regulated kinases; MAPK, mitogen-activated protein kinase.

Endothelial dysfunction is characterized by the impaired capacity to form new blood vessels. The development of new vasculature occurs through sprouting of endothelial cells from precedent blood vessels, a process known as angiogenesis. Angiogenesis is a critical process occurring in embryo and in adult throughout the lifetime, having a pivotal role in physiology and pathologies. Physiologically, it has a paramount role in important processes such as wound healing and muscle hypertrophy. Moreover, several pathological conditions share a common feature of impaired angiogenesis such as inflammatory diseases, cardiovascular disorders and atherosclerosis [86]. Among the regulators of blood vessels’ formation is vascular endothelial growth factor (VEGF) family members, having a primary role in the mediation of angiogenesis. VEGF is a large family of proteins including several members, VEGF-A, VEGF-B, VEGF-C, VEGF-D and the placental growth factor. VEGF-A is the most distinguished member owing to its potent stimulatory actions on angiogenic signaling. VEGF-A mediates its actions by binding to its receptor (VEGFR). VEGFR has three subtypes VEGFR 1, VEGFR 2 and VEGFR 3, where VEGFR 2 is the main receptor relying the signal and is widely expressed in endothelial cells and VSMCs. Binding of VEGF-A to VEGFR activates the intrinsic tyrosine kinase and ensuing auto-phosphorylation of the receptor (Figure 5), which acts as a docking site for several signaling molecules initiating subsequent phosphorylation and activation of downstream signaling cascades [86,87].

In order for angiogenesis to occur, endothelial cells must undergo migration and proliferation. A group of endothelial cells, known as tip cells lead the invasion into the surrounding tissue with the help of another group of endothelial cells known as trailing stalk cells that orchestrate the growth towards the source of VEGF-A stimuli. VEGF-A stimulates the migration of endothelial cells through activation of p38 MAPK while it stimulates proliferation by the activation of extracellular signal-regulated kinase 1/2 (ERK1/2) MAPK through phospholipase C_γ_/protein kinase C (PKC). VEGF-A also initiates migration through activation of Src, a protein tyrosine kinase, resulting in the phosphorylation of the junctional protein VE-cadherin. Importantly, VEGF-A also contributes in promoting endothelial cell survival through PI3-K/Akt pathway [88]. As VEGFR is tyrosine phosphorylation-dependent, this makes it a target for PTP1B. In vitro deletion of PTP1B in mouse primary endothelial cells resulted in the enhancement of VEGF-A-induced ERK activation and VEGFR2 phosphorylation. In correlation, cells displayed enhanced proliferation and accelerated tube formation capacity on a Matrigel assay. In vivo, endothelial-specific deletion of PTP1B enhanced endothelial cells sprouting in mouse retinas as well as increased the number of tip cells, indicating an improved angiogenesis. In accordance with improved angiogenesis, endothelial-PTP1B^−/−^ mice had a significantly faster wound healing compared to control mice [89]. Furthermore, mice lacking PTP1B were at a lower risk of developing CHF following experimental induction of myocardial infarction. This beneficial effect is brought about by improved endothelial cell migration and proliferation in response to VEGF-A, mediated through increased phosphorylation of VEGFR and VE-cadherin. Altogether, these results indicate improved angiogenesis and enhanced cardiac muscle perfusion in the absence of active PTP1B [90]. 

Correlating with the cardio-protective effect of PTP1B deletion, PTP1B^−/−^ mice fed a HFD were protected against HFD-induced cardiac anomalies when compared to their control counterparts. HFD-fed control mice had an increased heart weight along with elevated lipid accumulation in the heart resulting from upregulation of genes responsible for fatty acid metabolism in the heart. They also presented an elevation in cardiac hypertrophy and pro-hypertrophic markers. All these HFD-induced effects were completely prevented in PTP1B^−/−^ mice [91]. Endothelial cell-specific PTP1B deletion in adult mice improved their survival and heart function after the induction of cardiac hypertrophy. Endothelial-PTP1B^−/−^ mice exhibited improved angiogenesis, VEGFR phosphorylation, elevated levels of total eNOS and a significant reduction in cardiac fibrosis [50]. 

One of the detrimental complications of diabetes and indicative of impaired endothelial function is diabetic wound healing. To assess the role of PTP1B in the wound healing process in diabetes, diabetes was induced in wild type and PTP1B^−/−^ mice by injecting streptozotocin followed by creating a skin wound and observing the wound healing process. Wild type diabetic mice had a delayed wound healing process accompanied by distorted collagen layout and slow neovascularization, while PTP1B^−/−^ diabetic mice had normal wound healing pace along with organized collagen structure and boosted neovascularization. In the same way, cultured human microvascular endothelial cells (HMECs) exposed to high glucose showed a considerably abolished tube-formation when stimulated with VEGF, which was restored to a great extent by inhibiting PTP1B. Furthermore, under these treatment conditions, VEGFR phosphorylation was blunted in HMECs treated with high glucose, while the inhibition of PTP1B markedly enhanced VEGFR phosphorylation. To further asses the effect of PTP1B on endothelial cell migration and proliferation, a scratch/migration assay was made in a monolayer of HMECs incubated either in normal or high glucose in the presence or absence of PTP1B inhibitors. High glucose exposure reduced HMECs migration and proliferation when compared to normal glucose group. On the other hand, PTP1B inhibition promoted HMECs proliferation and migration even in the presence of high glucose [92].

#### 3.3.3. Endoplasmic Reticulum Stress as a Possible Bridging Link Between PTP1B, Insulin Resistance and Cardiovascular Dysfunction

PTP1B is located on the cytosolic face of the ER, and this critical location was reported to play a key role in ER stress response. Therefore, the relationship between ER stress and PTP1B has emerged as a novel level of molecular link behind the onset and development of endothelial dysfunction in obesity and diabetes.

The rough ER is an important intracellular organelle that controls synthesis, folding and post-translational modifications of proteins and its function is very tightly regulated. The ER mediates its functions through the action of several foldases and chaperones such as glucose regulated protein 78 (GRP78), to achieve proper folding of proteins and prevent their aggregation [13,14]. When the demand for protein synthesis and folding exceeds the capacity of the ER to fold proteins, a situation of stress is activated leading to the activation of unfolded protein response (UPR), which initially aims at recovering ER homeostasis and proper function. The UPR operates by (a) inhibiting general protein translation to reduce the load on the ER, (b) enhancing the ER folding capacity by boosting the synthesis of molecular chaperones and (c) activating ER-associated degradation machinery (ERAD) to get rid of irreversibly damaged proteins. However, when UPR fails to resume ER normal function and becomes chronically active, a state of ER stress is activated that is characterized by cellular inflammation and cell death [13,14].

The UPR mediates its actions via three main ER transmembrane effectors; protein kinase-like endoplasmic reticulum kinase (PERK), activating transcription factor (ATF)-6 and inositol requiring enzyme (IRE)-1α. The luminal domains of these effectors interact with chaperone GRP78 under unstressed conditions, which maintain them as inactive. The accumulation of unfolded or misfolded proteins within the ER lumen leads to the dissociation of GRP78 from UPR effectors, which subsequently become active [13,14]. Active PERK phosphorylates and hence activates eukaryotic initiation factor (eIF)-2α. Phosphorylated eIF-2α will then reduce general protein translation; however, it selectively enables the translation of ATF-4 that stimulates the transcription of a pro-apoptotic gene, CCAAT/enhancer-binding protein homologous protein (*CHOP*) [13,14]. ATF-6 freed from GRP78, moves to the Golgi apparatus where it is activated by proteolytic cleavage, and then translocates to the nucleus and stimulates the transcription of expression of several chaperones including GRP78 to further improve protein folding. If UPR fails to improve ER homeostasis, the cell may activate an apoptotic signaling response where ATF-6 can enhance the expression of CHOP expression in concert with ATF-4 [93]. IRE-1α possesses protein kinase and endo-ribonuclease activities. Active IRE-1α allows the splicing of X-box binding protein (*XBP*)-1 into an active variant, which stimulates the transcription of chaperones and components of the ERAD system to recover ER homeostasis. However, prolonged IRE-1α activation, stimulates apoptotic signaling pathways including p38 MAPK and c-Jun N-terminal Kinase (JNK) [94].

Chronic ER stress response activation has been closely associated with both insulin resistance and cardiovascular dysfunction particularly endothelial dysfunction (for a detailed review see [13,14]). Work by Özcan et al. [95] reported the activation of ER stress response in genetic and HFD mouse models of obesity [95]. Under ER stress conditions, prolonged activation the IRE-1α arm stimulates JNK which ultimately phosphorylates IRS-1 on serine residues causing its inhibition and hence a weaker insulin signaling response [96]. The expression of ATF-4 stimulates the expression of Tribbles homolog 3 (TRB3), a kinase known to inhibit Akt, leading eventually to further impairment of insulin signaling cascade [54]. Several studies have demonstrated the implication of the ER stress response in endothelial cell dysfunction. Endothelial cells derived from arterial areas with the highest risk of developing atherosclerotic plaques had chronic activation of ER stress markers [97,98]. Induction of ER stress in endothelial cells caused a concomitant increase in the expression of vasoconstrictor ET-1 and a reduction in eNOS expression [99]. Furthermore, the role of ER stress in atherosclerosis was highlighted using ApoE deficient mice (ApoE^−/−^) as a murine model for atherosclerosis, where the plaque size was reduced in aortas obtained from ApoE^−/−^ mice treated with chemical chaperones to alleviate ER stress [100]. Work by Tufanli et al. [101] reported that the treatment of macrophages with a selective inhibitor of IRE-1α reduced the expression of multiple genes involved in atherogenesis caused by ER stress in addition to reducing plaque size and improving collagen content in aortas from ApoE^−/−^ mice [101].

Several studies have harnessed the link between the ER stress response and PTP1B. Mouse embryonic fibroblasts (MEF) isolated from PTP1B^−/−^ and wild type mice were treated with azetidine-2-carboxylic acid (Azc) to induce ER stress. In wild type MEF, ER stress induction significantly increased the levels of phosphorylated JNK and p38 MAPK while their levels were undetectable in MEF from PTP1B^−/−^ mice. To further link PTP1B to the activation of these stress kinases, PTP1B^−/−^ MEF were exposed to retroviral infection to re-express PTP1B, which completely restored phosphorylation levels of both JNK and p38 MAPK in response to ER stressor Azc. Since JNK is activated through the action of UPR arm IRE-1α, this may indicate the implication of PTP1B in IRE-1α signaling. Further in support of this notion, spliced *XBP-1* mRNA levels were significantly downregulated in PTP1B^−/−^ MEF in comparison to wild type, despite similar expression levels of total unspliced *XBP-1* in both groups [102]. Since JNK is activated through the action of IRE-1α and JNK is pro-apoptotic, we could speculate that PTP1B may aggravate ER stress-induced apoptosis through IRE-1α signaling pathway. Further validating these results, in vivo induction of ER stress in liver-PTP1B^−/−^ mice failed to increase the levels of phosphorylated JNK and p38 MAPK. Similarly, liver-PTP1B^−/−^ mice exhibited reduced mRNA levels of pro-apoptotic *CHOP* and *XBP-1* in addition to protein expression levels of PERK and p-eIF-2α compared to controls [70].

To assess the impact of PTP1B on different branches of ER stress response, ER stress was induced in wild type and liver-PTP1B^−/−^ mice followed by the measurement of the expression of all three branches, IRE-1α, PERK and ATF-6, in addition to their downstream components. Liver-specific deletion of PTP1B resulted in the reduction of all ER stress branches and their components; (a) decreased IRE-1α activation and splicing of *XBP1*, (b) decreased expression of PERK, p-eIF-2α, ATF-4 and *CHOP* and (c) reduced expression of full-length ATF-6 and decreased ATF-6 cleavage. Furthermore, murine hearts exposed to tunicamycin, a pharmacological ER stressor, were protected from ER stress-induced anomalies by PTP1B deletion [103]. Altogether, these results indicate the importance of PTP1B in the full induction of ER stress response. Interestingly, there seems to be a reciprocal relationship between PTP1B and ER stress. The treatment of HepG2 hepatocyte cell line with tunicamycin caused a significant elevation in PTP1B mRNA and protein expression, indicating a possible cross-talk between ER stress and PTP1B [71]. Similar results were observed in the pancreas of mice where ER stress was induced by HFD [104]. Contrariwise, PTP1B deletion in adipose tissue did not provide the same beneficial effect on ER stress as with PTP1B deletion in liver. Adipose-PTP1B^−/−^ potentiated UPR response as evidenced by the upregulated expression of key ER stress markers [105].

To further investigate the crosstalk between ER stress and PTP1B, the basal levels of PTP1B were first assessed in mouse myotubes, followed by their treatment with tunicamycin. Tunicamycin successfully induced ER stress as demonstrated by increased levels of GRP78 in addition to significantly increasing PTP1B expression. This increase in expression was blunted when cells were treated with tauroursodeoxycholic acid (TUDCA), a chemical chaperone known to reduce ER stress response, suggesting that ER stress upregulated the expression of PTP1B. Simultaneously, the silencing of PTP1B failed to decrease the expression of the ER stress marker, GRP78, while it inhibited the phosphorylation of eIF-2α and JNK, suggesting a role for PTP1B in mediating PERK and IRE-1α signaling pathways [106].

Prolonged ER stress is known to cause detrimental health conditions and its complex interplay with PTP1B makes PTP1B as a potential target for a wide array of diseases including diabetes and underlying cardiovascular disorders.

## 4. PTP1B as a Potential Therapeutic Target

The implication of PTP1B in several pathological conditions, varying from metabolic disturbances to cancer, has placed PTP1B under the spotlight as being a promising therapeutic target [107]. However, every way to success is accompanied with challenges. Despite the tremendous efforts in the pursuit of designing selective PTP1B inhibitors, several challenges were faced. Initially, the strategy was to target the catalytic site using a phosphotyrosine mimetic small molecules, which faced two major challenges. The PTP family is a very closely related family having a highly conserved catalytic domain among family members. Inhibition by targeting the catalytic domain of PTP1B resulted in the inhibition of other family members, which may lead to significant undesirable off target side effects. For instance, PTP1B shares an overall similarity with its most closely related classical PTP, T cell protein tyrosine phosphatase (TC-PTP), by 72%. Furthermore, PTP1B and TC-PTP are 94% similar with regards to their catalytic sites [108,109]. PTP1B-deficient mice showed improved insulin sensitivity and resistance to HFD-induced obesity [16,61], while on the other hand, TC-PTP-deficient mice died in early life due to increased overall inflammation and severe anemia [110]. This indicates the need for highly selective PTP1B inhibitors. The second challenge faced was bioavailability and cell permeability. To target the catalytic site, phosphotyrosine mimetic small molecules were used, which was challenged by the fact that these molecules were positively charged having a high charge density, which restricted their cellular permeability [111].

To overcome these challenges, several approaches were used among which included targeting less conserved sites, the allosteric site as well as targeting side pockets present at the borders of the catalytic pockets. Such inhibitors are known as bidentate ligands, which are designed to bind not only to the catalytic site but to an allosteric site or side pockets as well. Such approach provides a potent inhibitor with enhanced selectivity towards PTP1B over other PTPs [112]. Based on that theory, a substance was used to target the C-terminus region of PTP1B, which was initially discovered in the liver of *Squalus acanthia*, dogfish shark. It is chemically known as trodusquemine (MSI-1436), and showed a very high selectivity in inhibiting PTP1B. HFD-induced obese mice that where treated with trodusquemine experienced a significant reduction in fat and insulin levels [113]. As trodusquemine showed promising effects, other derivatives to it are being developed including claramine, which showed similar actions as trodusquemine in addition to being easier than trodusquemine to synthesize [29,114]. Similarly, DPM-1001 is a recently reported analogue of trodusquemine, as being a potent and most importantly orally available selective PTP1B inhibitor [115]. Despite these efforts, none of the available inhibitors have made their way through to Phase II of the clinical trials yet.

Several identified synthetic small molecules can pave the way towards developing more efficient and improved inhibitors. Such molecules include the first discovered PTP1B inhibitor Difluoromethylene phosphonates, a heterocyclic carboxylic acid and vanadate-based phosphotyrosine mimetics [116]. Moreover, several marine natural compounds were isolated from marine organisms such as sponges, algae, marine fungi and soft corals that possess PTP1B inhibitory properties, making them promising lead compounds in the pursuit of PTP1B inhibitors that may overcome the presented challenges [117]. Genetic targeting of PTP1B was also employed. Swarbrick et al. [118] investigated the effect of inhibiting of PTP1B using antisense oligonucleotides (ISIS 113715) in monkeys. ISIS 113715 improved insulin sensitivity and caused a rise in adiponectin levels [118]. This compound has completed Phase II clinical trials (NCT00330330). Most recently, a new PTP1B inhibitor, XWJ24, was discovered, which unlike its parent drug Norathyriol is a potent inhibitor. XWJ24 inhibits PTP1B competitively with 4.5 fold selectivity over TC-PTP making it a highly promising compound for further modification and development [119].

## 5. Conclusions

PTP1B is involved in several pathological processes including diabetes and cardiovascular disease. Unlike existing insulin sensitizing strategies, the inhibition of PTP1B action was reported to improve glucose metabolism and enhance the insulin response without increasing lipid metabolism and causing lipid accumulation in livers. This represents an advantage over existing anti-diabetic pharmacological approaches and would prevent side effects related to body weight gain and liver steatosis. The role that PTP1B plays in the onset of cardiovascular disturbances including, endothelial dysfunction, cardiac function impairment and aberrant angiogenesis, also places this enzyme as a very attractive single therapeutic target for cardiovascular disorders. However, because of its ubiquitous expression and its involvement in multiple physiological processes, targeting PTP1B systemically may cause undesirable off target side effects that may limit its future clinical use. Therefore, in addition to the need for overcoming the challenges inherent to synthesizing potent and selective small molecule inhibitors for PTP1B, it is crucial to also develop novel approaches to specifically target tissues of interest such as cardiovascular system or insulin-sensitive tissues to optimize the clinical benefits of inhibiting PTP1B while reducing the risk of potential off target side effects.

## Figures and Tables

**Table 1:** Detrimental effects of obesity.

**Insulin Resistance**
Impaired glucose tolerance
Type-2 diabetes
**Abnormal Plasma Lipids**
High total cholesterol
Hypertriglyceridemia
High apolipoprotein B
Lower levels of apolipoprotein A1
**Hemodynamics**
Increased blood volume
Elevated LV wall stress
High arterial pressure
Pulmonary artery hypertension
**Structure of the Heart**
Remodeling of LV
Hypertrophy of LV
Hypertrophy of RV
Enlargement of LA
**Cardiac Function**
LV systolic and diastolic dysfunction
Failure of RV
**Inflammatory Response**
High levels of C-reactive protein
Overproduction of tumor necrosis factor (TNF)-α
**Neuro-Hormonal**
Hyperinsulinemia
Resistance to leptin and hyperleptinemia
Reduced adiponectin
Sympathetic activation

LV; left ventricle, RV; right ventricle, LA; left atrium.

**Table 2:** Major factors released by endothelial cells.

**Vascular Tone**	
**Endothelium derived relaxing factors**	**Endothelium derived contracting factors**
Nitric Oxide (NO)	Elevated triglyceride
Prostacyclin	Decreased apolipoprotein-A1
Bradykinin	Thromboxane
Endothelium-derived hyperpolarizing factor	
**Coagulation**	
**Anti-coagulants**	**Pro-coagulants**
Thrombomodulin	Von Willebrand factor
Protein C	Factor V
Urokinase	Plasminogen activator inhibitor
Tissue plasminogen activator	Tissue factor
**Adhesion and Permeability**	
Vascular cell adhesion molecule 1 (VCAM-1)	Tumor necrosis factor-α (TNF-α)
Intracellular adhesion molecule 1 (ICAM-1)	Monocyte chemoattractant protein 1 (MCP-1)
Platelet-endothelial cells adhesion molecule	Cytokines
**Differentiation and Cellular Growth**	
Transforming growth factor-β (TGF-β)	Insulin-like growth factor 1 (IGF-1)
Platelet-derived growth factor (PDGF)	Basic fibroblast growth factor (BFGF)
